# Volatile Changes during Storage of Shelf Stable Apple Juice: Integrating GC-MS Fingerprinting and Chemometrics

**DOI:** 10.3390/foods9020165

**Published:** 2020-02-10

**Authors:** Biniam Kebede, Vivien Ting, Graham Eyres, Indrawati Oey

**Affiliations:** 1 Department of Food Science, University of Otago, PO BOX 56, Dunedin 9054, New Zealand; vivientsy21@gmail.com (V.T.); graham.eyres@otago.ac.nz (G.E.); indrawati.oey@otago.ac.nz (I.O.); 2 Riddet Institute, Private Bag 11 222, Palmerston North 4442, New Zealand

**Keywords:** apple juice, volatiles, shelf life, fingerprinting, chemometrics, chemical reactions

## Abstract

This is the first study to reveal potential markers for volatile changes during ambient and accelerated shelf life of pasteurized apple juice. The volatile changes were monitored at 20, 30 and 40 °C using a headspace solid-phase microextraction-gas chromatography-mass spectrometry fingerprinting method. Using modern chemometrics and feature selection, hexanal, trans-2-hexenal, dimethyl sulphide, furfural, ethyl acetate and 1-pentanol were chosen as potential shelf life markers. Volatiles associated with the green, grassy and fresh apple aroma, such as hexanal and trans-2-hexenal, decreased during storage, whereas thermal load and browning associated compounds, like dimethyl sulphide and furfural, increased during storage. Hexanal and trans-2-hexenal can be markers to monitor the change in green-apple like character. Furfural and dimethyl sulphide can be markers of temperature abuse during juice processing and storage. Furfural can also be an indicator for juice browning. The present work effectively identified potential markers to monitor and predict volatile aroma changes of shelf stable apple juice in different storage conditions. Sensory analysis can be conducted in the future to confirm the aroma relevance of selected markers.

## 1. Introduction

Aroma is one of the most important quality attributes of an apple, which is mainly attributed to its volatile profile. Hundreds of volatile compounds have been detected in the aroma profile of apples. These compounds are categorized as esters, alcohols, aldehydes, ketones and ethers [1,2]. Some of these compounds are labeled as “character impact,” including trans-2-hexenal (green), hexanal (grassy, green), butyl acetate (red apple aroma), ethyl butanoate (fruity), butan-1-ol (sweet aroma), etc. [3]. 

A large quantity of harvested apples can be processed into a juice. Thermal processing, using the high temperature short time principle, is still the most common method of preservation for an apple juice. This process results in a shelf stable product with a shelf life up to several months (when stored under the recommended conditions). Quality-related (bio)chemical reactions triggered during juicing and/or processing will continue during the storage time, thus the aroma of apple juice will further change during shelf life. Specifically, reactions such as oxidation and Maillard could lead to the loss of the fresh apple juice aroma and/or formation of off-odor or cooked-note during shelf life. However, based on our knowledge, no study could be found in literature investigating volatile changes during ambient and accelerated shelf life of pasteurized apple juice. Very few studies are available investigating the volatile changes during refrigerated shelf life of minimally processed apple juice [4,5]. Therefore, there remains a need for a method and a chemical marker, which would serve as a useful index of volatile aroma changes during ambient and accelerated shelf life of pasteurized apple juice. 

Numerous studies have focused on fresh apple juice flavor profiles [6,7,8]; comparing volatile profile of different cultivars [9,10]; effect of harvest date on volatiles compounds [11] and the influence of processing on the aroma [5,12,13,14]. However, no studies investigated volatile aroma changes during ambient and accelerated shelf life conditions of processed apple juice. In that framework, the aim of this study was to implement a gas chromatography-mass spectrometry (GC-MS) fingerprinting approach to track volatile changes in pasteurized apple juice during storage and determine reliable shelf life markers/indices. As a case study, a New Zealand Royal Gala apple was chosen. After thermal pasteurization, juices were stored at 20, 30 and 40 °C to set-up accelerated shelf life testing (ASLT). A headspace solid-phase microextraction-gas chromatography-mass spectrometry (HS-SPME-GC-MS) fingerprinting methodology was implemented to detect a wide range of volatile compounds in the shelf stable apple juice headspaces. SPME is one of the most commonly used extraction techniques due to its sensitivity and selectivity and solvent-free nature. In this work, SPME using a divinylbenzene/carboxen/polydimethylsiloxane (DVB/CAR/PDMS) fibre coating was chosen due to its proven ability to detect a wide range of volatile compounds [15,16,17,18,19]. The obtained volatile fingerprinting data was analysed using advanced chemometrics methods. The state-of-the-art chemometrics and feature selection techniques [16,17,18,19] were used to track the volatile changes during storage and identify potential shelf life markers.

## 2. Materials and Methods

### 2.1. Sample Preparation, Thermal Pasteurization and Stroage

A single batch of Royal Gala apple was obtained from Mill’s Orchard, Rangiora, New Zealand. After juicing, ascorbic acid (500 mg/L) was added into the apple juice to minimize enzymatic browning. The juice was then pasteurized at 86 °C for 22 s (HIPEX UHT/Pasteurizing Pilot Plant, FoodSouth, Christchurch, New Zealand) and aseptically packed into sterile aluminum pouches (1 L/bag) via an Engi-O aseptic filler. Pasteurized shelf stable juices were then stored at three temperatures (20, 30 and 40 °C) for up to 16 weeks to set up an ASLT. At fixed points in time (a maximum of eleven at each storage temperature), stored bags were sampled from the incubators. The detailed sampling plan can be found in the supplementary material (see Table S1). Prior to opening, the bags were cooled at 4 °C for 2 h. Thereafter, samples were frozen in liquid nitrogen and stored at −40 °C. All stored samples were analyzed together in a single batch after the completion of the shelf life. 

### 2.2. Headspace Gas Chromatography-Mass Spectrometry (GC-MS) Fingerprinting Analysis 

Headspace-solid phase microextraction (HS-SPME) procedure was conducted according to Buvé, Neckebroeck, Haenen, Kebede, Hendrickx, Grauwet and Van Loey [18] with some modifications. Frozen samples were gently thawed overnight in a cooling room at 4 °C. An aliquot of 8 mL apple juice was added into a 20 mL GC glass vial with polytetrafluoroethylene (PTFE) /silicone septum and crimp caps. Volatile compounds were extracted using headspace-solid phase micro-extraction (HS-SPME) method, and analysed using the Agilent 6890N GC system connected to an Agilent MSD 5975 VL. Equilibration, extraction and desorption of volatile compounds were automated using an auto sampler. The juice in the vial was equilibrated at 40 °C for 5 min. Next, headspace volatiles were extracted using an SPME fiber with a 50/30 µm divinylbenzene/carboxen/polydimethylsiloxane (DVB/CAR/PDMS) polymer at 40 °C for 30 min. The fibre was conditioned and cleaned as recommended by the suppliers. Four independent SPME extractions were performed for each sample. Following extraction, the volatile compounds were thermally (230 °C during 5 min) desorbed at the GC-inlet in a splitless mode. Separation was done using Zebron ZB-Wax column (60 m × 0.32 mm inner diameter × 0.5 µm film thickness; Phenomenex) at a constant flow rate of 1 mL/min. The GC oven temperature was initially held at 50 °C for 5 min, followed by heating at 5 °C/min to reach 210 °C, and then further ramped at 10 °C/min to reach the final 240 °C and maintained for the next 5 min before cooling to 50 °C. Electron ionisation at 70 eV was used to obtain the mass spectra with a scanning range of 35 to 400 m/z. The mass spectrometry ion source and MS quadrupole temperature was set at 230 °C and 150 °C, respectively. In the present work, samples were analyzed in a randomized fashion. Furthermore, day 0 (unstored) juice samples were injected into every 10 GC-MS analyses as a quality control (QC) sample. Blank samples were also used to monitor any carryover and integrity of the fibre.

### 2.3. Chemometrics Data Analysis

#### 2.3.1. Data Pre-Processing: From Chromatogram to Data Table

As described by Kebede, Grauwet, Tabilo-Munizaga, Palmers, Vervoort, Hendrickx and Van Loey [16], Kebede, et al. [20] and Buvé, Neckebroeck, Haenen, Kebede, Hendrickx, Grauwet and Van Loey [18], the raw GC-MS total ion chromatogram was processed using automated mass spectral deconvolution and identification system (AMDIS, version 2.72, 2014, Agilent Technologies, USA) and mass profiler professional (MPP, version 14.9.1, 2017, Agilent Technologies, Australia) software. AMDIS was used to deconvolute peaks. MPP was then applied to filter out non-reproducible and irregular peaks and to align peaks. 

#### 2.3.2. Chemometrics and Marker Selection

The data obtained from MPP was analyzed using chemometrics data analysis which was carried out in Solo (Version 6.5, 2018, Eigenvector Research, Wenatchee, WA, USA). As discussed by Kebede, et al. [21] and Buvé, Neckebroeck, Haenen, Kebede, Hendrickx, Grauwet and Van Loey [18], two types of chemometrics techniques were employed: unsupervised principal component analysis (PCA) and supervised partial least squares regression (PLSR). The data was mean centered and scaled (weighed by their standard deviation). The unsupervised PCA was used to explore the data for any patterns and groupings and also to detect any outliers. Following that, PLSR was applied to study the evolution of volatiles in the headspace fraction during storage. For PLSR, the detected volatiles were considered as *X*-variables and storage time was considered as *Y*-variable. The optimum number of latent variables (LVs) for the model was selected using cross validation. To avoid the risk of overfitting, LVs that explain the maximum variance within the data at the minimum noise (root mean squared error; RMSE) were selected based on the applied cross validation. Bi-plots were constructed to visualize the volatile changes during storage. In order to select compounds significantly changing during storage (potential shelf life markers), variable identification (VID) coefficients were calculated. VID estimates the correlation coefficient between the detected volatiles and storage time. To identify compounds significantly affected by storage time, volatiles with an absolute VID value higher than 0.750 were selected and identified. To confirm the identity, three criteria were employed: (a) matching retention time and spectra with injected reference standards for at least one volatile from each chemical class; (b) comparison of the experimental retention index (RI) with RI according to literature; (c) match with the NIST library (NIST14, version 2.2, National Institute of Standards and Technology, Gaithersburg, MD, USA) of not less than 90% fit quality. For the RI calculation, alkanes were injected on the same ZB-Wax column and a maximum deviation of < 30 was used to support compound identifications. 

## 3. Results and Discussion 

### 3.1. Analyzing Volatile Changes during Storage of Shelf Stable Apple Juice

**Figure 1:**
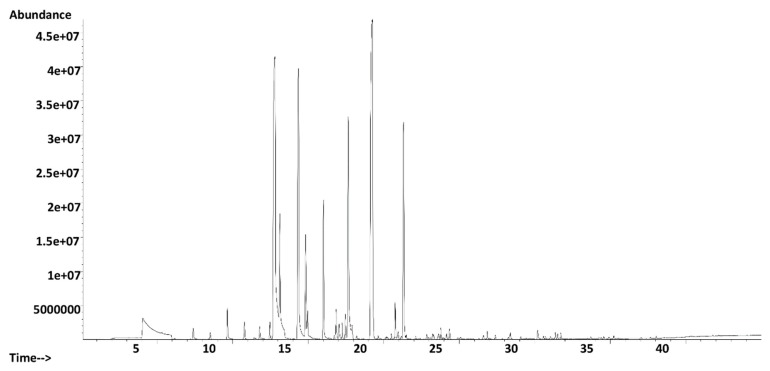
An exemplary total ion chromatogram of a volatile fraction of pasteurized apple juice obtained by the headspace solid-phase microextraction-gas chromatography-mass spectrometry (HS-SPME-GC-MS) fingerprinting method.

A representative total ion chromatogram of pasteurized apple juice obtained with the HS-SPME–GC–MS fingerprinting is depicted in Figure 1. The fingerprinting method enabled a detection of 70 volatile compounds in the pasteurized apple juice headspace, including esters, aldehydes, alcohols, carboxylic acid, ketones, etc. Comparable volatile compounds chemical groups have been reported in several apple cultivars [3,22,23]. 

The data was analyzed using chemometrics software called Solo (Version 6.5, 2018, Eigenvector Research, Wenatchee, WA, USA). The data was mean centered and scaled (weighed by their standard deviation). The unsupervised PCA was used to explore the data for any patterns and groupings and to detect outliers. In this work, a PCA model using three principal components showed a clear trend in all storage temperatures. As can be seen from the PCA bi-lots (Figure S1), the headspace fraction of the pasteurized apple juice changed as a function of storage time (from day 0 to week 16) and the trend was slightly clearer at higher storage temperatures. Furthermore, no outliers could be detected using PCA.

**Figure 2:**
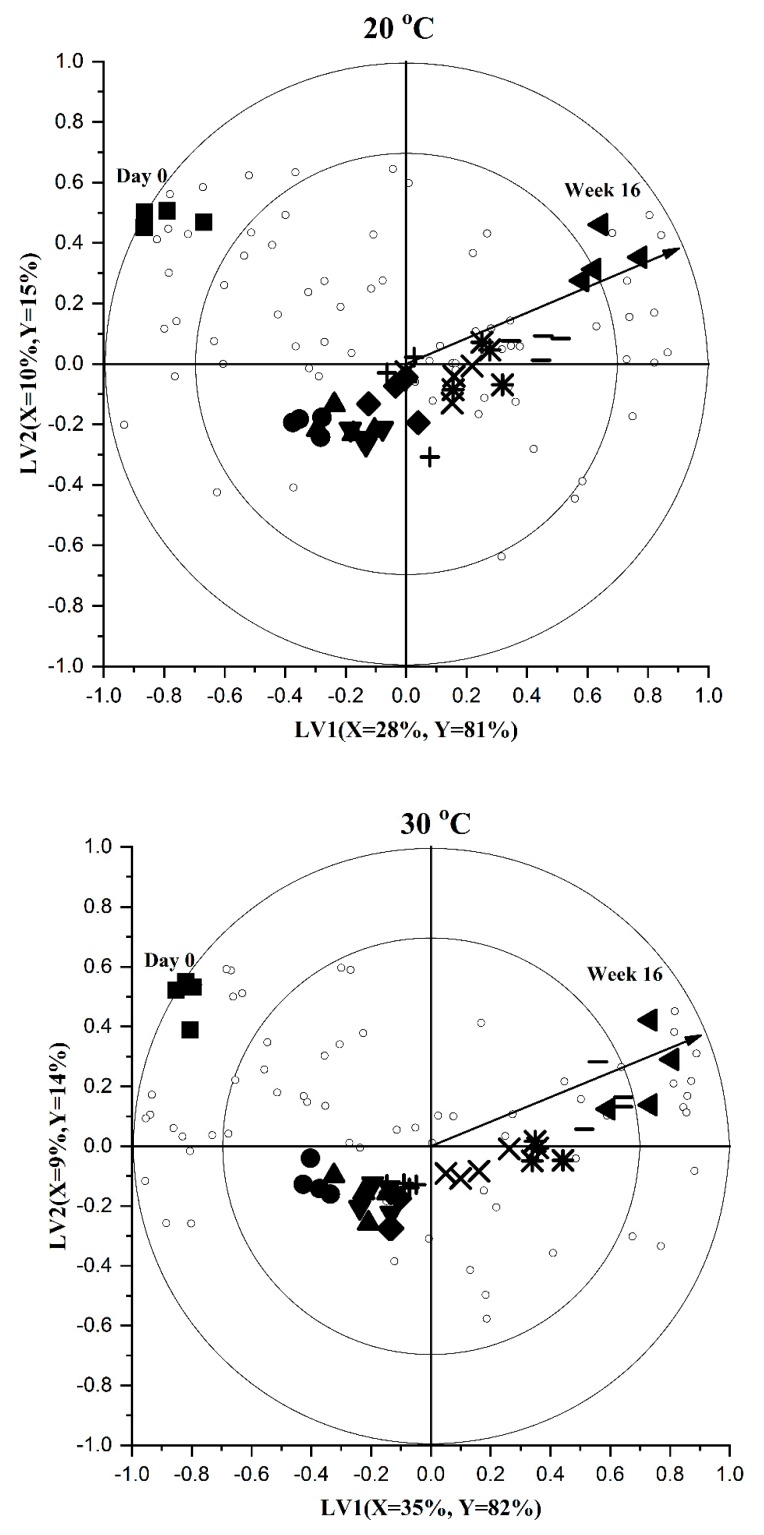

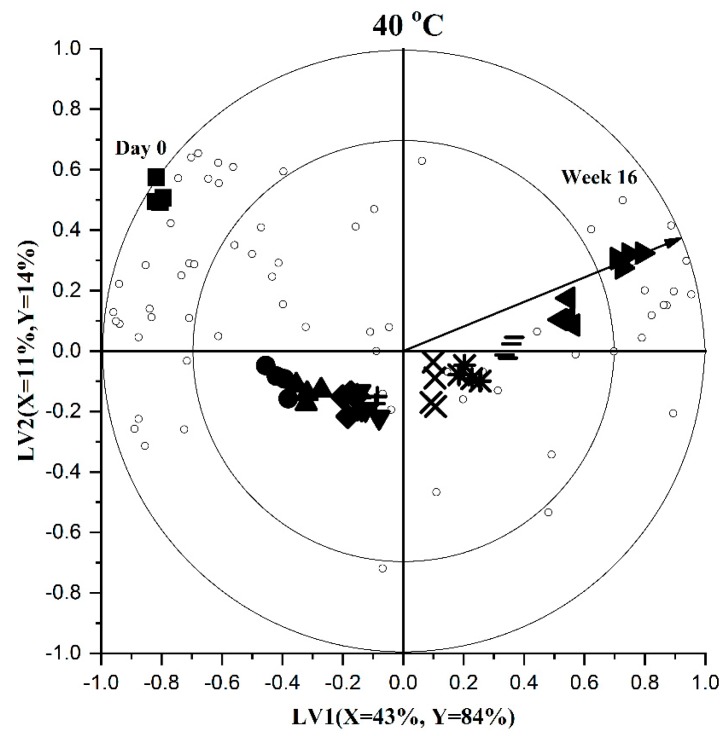
Partial least squares regression (PLSR) bi-plots visualising volatile changes in the headspace fraction of pasteurized apple juice during storage at 20, 30 and 40 °C. Up to 11 sampling points were used during storage at each storage temperatures (details are attached in the supplementary): time 1 (■); time 2 (●); time 3 (▲); time 4 (▼); time 5 (♦); time 6 (**+**); time 7 (X); time 8 (*); time 9 (-); time 10 (◀); time 11 (►). The *X* and *Y*-variances represented by latent variables (LV)1 and LV2 are shown.

PLSR was applied to further study the evolution of volatiles in the headspace fraction during storage and determine reliable shelf life markers. In PLSR, both the *X*-variables (detected volatile compounds) and *Y*-variable (storage time) data are considered (in PCA, only *X*-variables are considered). The goal of a PLSR analysis was to correlate volatile compounds and storage time using a linear multivariate model. Cross validation was used to choose the optimum number latent variables (LVs) for the PLSR that maximally describe the variation with a minimum noise. Using contiguous blocks cross validation, two LVs were selected as optimum, explaining more than 95% of the *Y*-variance in all storage temperatures. Based on the models, three bi-plots, one for each temperature, were constructed to visualize the volatile changes during storage (Figure 2).

These bi-plots show a comparable trend for the PCA plots, in which there is a clear effect of storage time on the apple juice volatile compounds at all storage temperatures. As discussed before, the trend seems to become slightly clearer at high storage temperatures indicating an effect of temperature in addition to storage time. On the bi-plots, the unfilled small circles represent the volatile compounds. Some of these compounds are positioned close to day 0 samples and others are projected towards the end of storage time. This seems to suggest that the concentration of some of the volatile compounds have decreased during storage while some other compounds appear to be formed/increased.

Furthermore, when closely examining the bi-plots, two trends can be seen. The first one is the horizontal projection of the samples from start (day 0) to the end (week 16) of storage time. This clear horizontal trend (variation) is represented by the first LV (> 80% of the *Y*-variance at all three storage temperatures). The second trend on the bi-plots is the V-shaped structure of the samples, which seems to be explained by the second LV. Such trends indicate the presence of possible complex (bio)chemical changes. In general, the bi-plots demonstrate that multiple complex degradation and formation reactions occur during storage of pasteurized apple juice. Comparable complex trends were previously reported when studying chemical changes during shelf life of pasteurized orange juice [24], pasteurized strawberry juice [18] and sterilized carrot purees [20]. 

The bi-plot is a powerful tool to graphically observe and interpret the evolution of volatiles as a function of storage time. However, the aim of this study was to identify volatile compounds affected by the storage time and temperature, which could be used as potential shelf life markers. Hence, feature selection was performed using a variable identification (VID) technique [17,18]. VID estimates the correlation coefficient between the detected volatile compounds and storage time. In other words, VID will enable the determination of headspace compounds significantly changing during storage. Aiming for compounds significantly affected by storage, variables with an absolute VID coefficient value higher than 0.750 were chosen (Table 1). These compounds were identified. 

### 3.2. Potential Markers of Aroma Changes during Storage of Shelf Stable Apple Juice

In samples stored at 20, 30 and 40 °C, respectively, 9, 16 and 17 volatile compounds were selected based on the VID technique. The selected compounds can be categorized as aldehyde, ester, sulphur compound, furan, alcohol and carboxylic acid chemical families. Most of these volatile compounds have been detected in the headspace of several apple varieties [3,22]. Aldehydes such as hexanal and trans-2-hexenal and most ester compounds were selected with negative VID coefficients, indicating the concentrations of these compounds have decreased during storage. On the other side, volatiles from sulphur compound, furan, alcohol and carboxylic acid chemical families have shown an increasing/formation trend as a function of storage time (selected with positive VID values).

When comparing the selected compounds among the 20, 30 and 40 °C storage temperatures, similar chemical groups were selected. This seems to suggest the presence of comparable reaction pathways at all storage temperatures. Moreover, more volatiles were selected at higher storage temperatures compared to the ambient condition. In the present work, two criteria were used for choosing potential shelf life markers/indices: (i) the peak area must change not only at higher storage temperature but also at the ambient condition (to verify the applicability for ASLT); and (ii) be characteristic aroma compounds in fresh and/or processed apple juice. Based on these criteria, hexanal, trans-2-hexenal, dimethyl sulphide, furfural, ethyl acetate and 1-pentanol were chosen as potential markers for volatile flavor changes during shelf life of processed apple juice. 

**Figure 3:**
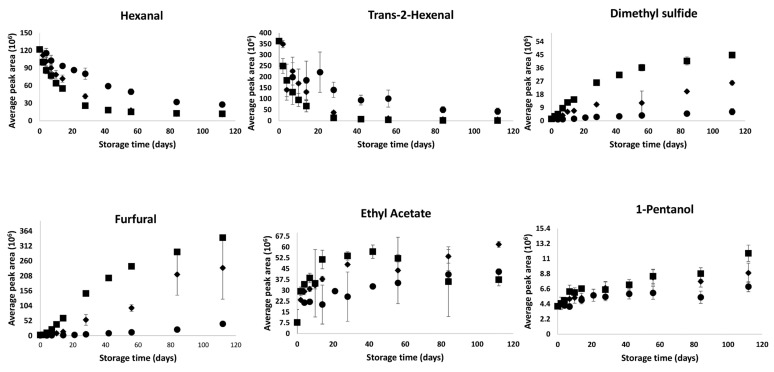
Average peak area of potential shelf life markers during storage in pasteurized apple juice stored at 20 °C (●), 30 °C (♦) and 40 °C (■). Standard deviation of 4 individual headspace extraction is included.

Figure 3 shows the evolution during storage of these potential shelf life marker compounds at the three storage temperatures. The peak area obtained after peak deconvolution using AMDIS was used to investigate the changes. The changes are influenced by both storage time and temperature. On the one hand, the amount of hexanal and trans-2-hexenal have decreased during storage. On the other hand, dimethyl sulphide, furfural, 1-pentanol and ethyl acetate have shown an increasing trend as a function of storage time. Buvé, Neckebroeck, Haenen, Kebede, Hendrickx, Grauwet and Van Loey [18] have also observed a decreasing trend for aldehydes such as hexanal, trans-2-hexenal and an increasing trend for furfural and dimethyl sulphide during shelf life for pasteurized strawberry juice. There is limited literature information reporting the changes in these compounds during shelf life of pasteurized apple juice. Nevertheless, to obtain an insight into the shelf life changes, the evolution of some of these markers was interpreted and discussed in relation to relevant (bio)chemical reactions based on available information on other citrus juices. 

Hexanal and trans-2-hexenal are associated with “green,” “grassy” and “fresh” character in several apple cultivars [3]. From Figure 3, at the start of storage these compounds seem to appear at higher amounts in pasteurized apple juice. During storage, however, the amounts of these compounds decreased to lower levels, possibly due to oxidative breakdown processes. This shows the significant effect of shelf life, and that process impact evaluations should not only be investigated immediately after processing but also during shelf life. Buvé, Neckebroeck, Haenen, Kebede, Hendrickx, Grauwet and Van Loey [18] have also reported a fast degradation of hexanal and 2-hexenal during storage of pasteurized strawberry puree. The loss of these compounds could result in the loss of the fresh, green and grassy note of the apple juice during storage. Sensory analysis needs to be conducted to confirm this hypothesis, but the output from this work shows that hexanal and trans-2-hexenal can be used as potential shelf life markers of the green-apple like character. 

The concentration of ethyl acetate has shown an increasing trend during storage. Previous studies have reported a decrease in the volatile release of trans-2-hexenal and an increase in acetate esters during enzymatic browning of apple juice. The authors have associated this trend to a decrease in green odour and an increase of sweet odour during browning [25]. A similar trend can be hypothesized in the present work. However, this needs to be further confirmed with a sensory analysis.

Furfural has been reported as an index of storage temperature abuse in commercially processed citrus juices [26]. In the present work the amount of furfural was low at the beginning of storage and has shown an increasing trend as a function of storage time and temperature. In citrus fruit juices, furfural is reported to stem from the decomposition of ascorbic acid. In literature, both aerobic and anaerobic degradation of ascorbic acid have been linked with furfural formation. The oxidative reaction involves degradation of ascorbic acid to dehydroascorbic acid, followed by hydration of dehydroascorbic acid to ketogluconic acid, and finally decarboxylation and dehydration to furfural. Non-oxidative degradation occurs under acidic conditions, in which ascorbic acid degrades into furfural and 3-deoxy-L-pentose [27,28]. Furthermore, furfural has been considered an important intermediate and indicator of browning of orange juices [29]. Hence, the present work indicates the potential of furfural to be used as an important shelf life index/marker of temperature abuse and browning of pasteurized apple juice.

Dimethyl sulphide is selected with a high VID coefficient at all storage temperatures, which shows that its amount has increased during storage time. The formation of dimethyl sulphide is associated with reactions involving sulphur containing amino acids, such as methionine. During high temperature processing and storage conditions, methionine breaks down and further oxidises into dimethyl sulphide [30]. This compound has been reported with a strong relation to the thermal load of processing and/or storage conditions. Wibowo, Grauwet, Kebede, Hendrickx and Van Loey [24], Buvé, Neckebroeck, Haenen, Kebede, Hendrickx, Grauwet and Van Loey [18] and Kebede, Grauwet, Palmers, Vervoort, Carle, Hendrickx and Van Loey [21] have also reported an increased formation of dimethyl sulphide during storage (above 20 °C) of pasteurized orange juice, pasteurized strawberry puree and sterilized carrot puree, respectively. As dimethyl sulphide can give rise to an unwanted cooked-note, conditions that can lead to temperature abuse should be minimized throughput the whole production and supply chain. The present work shows that dimethyl sulphide can be used as a reliable shelf life indicator of temperature abuse during storage of processing apple juice.

1-Pentanol is another volatile showing an increasing trend during storage. This increasing trend is affected by temperature, but at a lower extent when compared to furfural and dimethyl sulphide. Contrary to furfural and dimethyl sulphide, 1-pentanol was already detected at a relatively higher amount at the beginning of storage (immediately after processing). This seems to suggest that the reactions responsible for this compound have already been triggered during juice and/or pasteurization steps. Possibly this formation could be due to an oxidative reaction triggered and continued during storage.

## 4. Conclusions

This study obtained an increased insight into volatile changes during storage (at 20, 30 and 40 °C) of shelf stable apple juice. Using HS-SPME-GC-MS fingerprinting, 70 volatile compounds could be effectively detected in pasteurized apple juice. Advanced chemometrics and feature selection techniques revealed potential markers for volatile aroma changes during storage. These compounds can be grouped under aldehyde (e.g., hexanal, trans-2-hexenal), sulfur compound (dimethyl sulphide), furans (furfural), ester (ethyl acetate) and alcohol (1-pentanol) chemical groups. Volatile markers associated with the grassy, green and fresh apple aroma (e.g., hexanal, trans-2-hexenal) seem to decrease during storage, whereas thermal-load (e.g., dimethyl sulphide) and browning (e.g., furfural) indicator compounds increased during storage. The selected compounds were also affected by the storage temperature. Furthermore, most of these compounds are odour-active volatiles. This seems to suggest their potential to be used as markers for accelerated shelf life testing of pasteurized apple juice. Hexanal and trans-2-hexenal can be potential markers to monitor the loss in green-apple like character. Furfural and dimethyl sulphide can be markers of temperature abuse. Furfural can also be an indicator for juice browning. The present work effectively identified potential markers/indices to monitor, optimize and predict volatile aroma changes of shelf stable apple juice in different storage conditions. In the future, the kinetics of selected shelf-life compounds should be studied in the context of accelerated shelf-life testing (ASLT). Furthermore, based on the obtained results, it is less straightforward to associate the changes in the selected markers to overall apple juice flavor. In the future, there is a need to conduct a sensorial analysis to understand how the observed volatile changes will be perceived. Furthermore, there is a need to investigate the volatile changes and selected markers in other apple cultivars. 

## Figures and Tables

**Table 1:** Headspace compounds significantly changing during storage at each 20 °C, 30 °C and 40 °C storage temperatures. Volatiles selected as potential shelf life markers/indices are put in bold. The retention index (RI) and chemical family are also listed. The identity of these compounds was further confirmed by injecting pure standard on the same GC-MS method.

Storage Temperature	VID	Identity	RI (Zebron ZB-Wax)	Chemical Group
**20 °C**	−0.953	**Hexanal**	1077	Aldehyde
	−0.751	**Trans-2-hexenal**	1242	Aldehyde
	−0.750	5-Hexenyl acetate	1357	Ester
	0.754	**1-Pentanol**	1263	Alcohol
	0.777	Hexanoic acid	1851	Carboxylic acid
	0.797	**Ethyl acetate**	866	Ester
	0.837	2,4-Di-tert-butylphenol	2143	Phenol
	0.901	**Furfural**	1508	Furan
	0.922	**Dimethyl sulphide**	790	Sulfur compound
**30 °C**	−0.959	**Hexanal**	1077	Aldehyde
	−0.919	(E)-2-hexen-1-ol	1434	Alcohol
	−0.914	5-Hexenyl acetate	1357	Ester
	−0.897	Trans-3-hexenyl acetate	1347	Ester
	−0.878	2-Hexen-1-ol, acetate	1364	Ester
	−0.838	3-Methyl-3-buten-1-ol, acetate	1209	Ester
	−0.831	**Trans-2-hexenal**	1242	Aldehyde
	−0.806	Butyl propionate	1144	Ester
	−0.792	Amyl acetate	1182	Ester
	0.838	Hexanoic acid	1851	Carboxylic acid
	0.846	**Ethyl acetate**	866	Ester
	0.853	2-Methyl-1-butanol	1213	Alcohol
	0.860	2,4-Di-tert-butylphenol	2143	Phenol
	0.875	**1-Pentanol**	1263	Alcohol
	0.891	**Furfural**	1508	Furans
	0.933	**Dimethyl sulphide**	790	Sulfur compound
**40 °C**	−0.923	3-Methyl-3-buten-1-ol, acetate	1209	Ester
	−0.913	Trans-3-hexenyl acetate	1347	Ester
	−0.911	5-Hexenyl acetate	1357	Ester
	−0.904	Isobutyl acetate	990	Ester
	−0.901	**Hexanal**	1077	Aldehyde
	−0.901	N-pPropyl acetate	949	Ester
	−0.876	2-Hexen-1-ol, acetate	1364	Ester
	−0.849	Amyl acetate	1182	Ester
	−0.793	3-(Methylthio)propyl acetate	1662	Ester
	−0.777	**Trans-2-hexenal**	1242	Aldehyde
	0.829	Hexanoic acid	1851	Carboxylic acid
	0.833	2-Octanone	1315	Hydrocarbon
	0.875	1-Bromo-2-methyl-cyclohexane	1281	Hydrocarbon
	0.885	2-Methyl-1-butanol	1213	Alcohol
	0.917	**1-Pentanol**	1263	Alcohol
	0.972	**Dimethyl sulphide**	790	Sulfur compound
	0.978	**Furfural**	1508	Furan

GC-MS: gas chromatography-mass spectrometry; VID: Variable identification.

## Supplementary Materials

The following are available online at https://www.mdpi.com/2304-8158/9/2/165/s1, Figure S1: PCA bi-plots showing volatile changes in pasteurized apple juice during storage at 20, 30 and 40 °C. The X-variances explained by each latent variable (LVs) are shown. Arrow is included to clearly show the trend as a function of storage time, Table S1: Details of shelf life planning. At fixed points in time (up to 11 time points per temperature), stored bags were sampled from the incubators.
