# Supplementary material for: Volatile Changes during Storage of Shelf Stable Apple Juice: Integrating GC-MS Fingerprinting and Chemometrics
Source: Foods. 2020 Feb 10;9(2):165. doi: 10.3390/foods9020165 (PMC7073669; doi:10.3390/foods9020165)
Supplement: Supplementary file 1 [file foods-09-00165-s001.pdf]

Supplementary Materials:

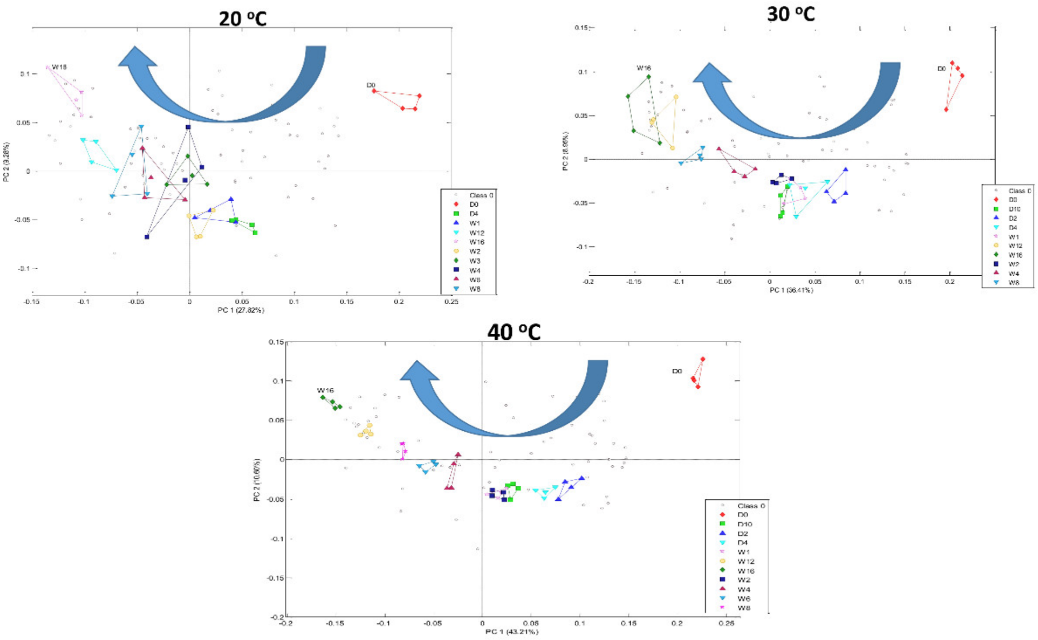

Figure S1. PCA bi-plots showing volatile changes in pasteurized apple juice during storage at 20, 30 and 40 °C. The *X*-variances explained by each latent variable (LVs) are shown. Arrow is included to clearly show the trend as a function of storage time.

Table S1. Details of shelf life planning. At fixed points in time (up to 11 time points per temperature), stored bags were sampled from the incubators.

	Days at 20 °C	Days at 30 °C	Days at 40 °C
Time 1	0	0	0
Time 2	4	2	2
Time 3	7	4	4
Time 4	14	7	7
Time 5	21	10	10
Time 6	28	14	14
Time 7	42	28	28
Time 8	56	56	42
Time 9	84	84	56
Time 10	112	112	84
Time 11			112
